# Fast ion conductivity in strained defect-fluorite structure created by ion tracks in Gd_2_Ti_2_O_7_

**DOI:** 10.1038/srep16297

**Published:** 2015-11-10

**Authors:** Dilpuneet S. Aidhy, Ritesh Sachan, Eva Zarkadoula, Olli Pakarinen, Matthew F. Chisholm, Yanwen Zhang, William J. Weber

**Affiliations:** 1 Materials Science and Technology Division, Oak Ridge National Laboratory, Oak Ridge, TN 37831; 2 Materials Science and Engineering, University of Tennessee, Knoxville, TN 37996.

## Abstract

The structure and ion-conducting properties of the defect-fluorite ring structure formed around amorphous ion-tracks by swift heavy ion irradiation of Gd_2_Ti_2_O_7_ pyrochlore are investigated. High angle annular dark field imaging complemented with ion-track molecular dynamics simulations show that the atoms in the ring structure are disordered, and have relatively larger cation-cation interspacing than in the bulk pyrochlore, illustrating the presence of tensile strain in the ring region. Density functional theory calculations show that the non-equilibrium defect-fluorite structure can be stabilized by tensile strain. The pyrochlore to defect-fluorite structure transformation in the ring region is predicted to be induced by recrystallization during a melt-quench process and stabilized by tensile strain. Static pair-potential calculations show that planar tensile strain lowers oxygen vacancy migration barriers in pyrochlores, in agreement with recent studies on fluorite and perovskite materials. In view of these results, it is suggested that strain engineering could be simultaneously used to stabilize the defect-fluorite structure and gain control over its high ion-conducting properties.

Besides interesting features such as frustrated magnetism^1^, ferroelectricity^2^, photocatalysis^3^, photoluminescence^4^, and giant magnetoresistence^5^, materials with the A_2_B_2_O_7_ pyrochlore structure have attracted significant interest as fast-ion conductors for electrolytes in solid oxide fuel cells^6^,^7^. The interest originates particularly due to the presence of one vacant site per eight oxygen sites that could provide fast channels for oxygen diffusion. However, the ordered arrangement of *A* and *B* cations locks most of these vacant sites allowing only their partial availability; while the sites can be unlocked by cation disorder leading to significantly enhanced oxygen diffusion, the disordered arrangement is generally not the ground state equilibrium structure in most of the pyrochlore materials^6^,^8^. Thus, achieving a stable disordered structure is one of the main bottlenecks. The formation of ion tracks by swift heavy ion irradiation^9^,^10^,^11^ is known to lead to cation disordering, and TEM investigations of such ion-tracks in Gd_2_Ti_2_O_7_ and other pyrochlores show that the disordered structure is present as a concentric ring region surrounding the amorphous core of the track^11^. Gaining control over the stabilization of the disordered structure via other more common synthesis techniques will depend on understanding the underlying features of the ring region. This understanding would also be crucial to the radiation-damage response of pyrochlore materials that are candidate materials for nuclear waste storage applications^12^,^13^,^14^.

In this work, we focus on the nature and properties of the concentric, defect-fluorite ring structure, which surrounds the amorphous track in Gd_2_Ti_2_O_7_ pyrochlore, by means of experimental observations and theoretical calculations, leading to the quantitative predictions of enhanced oxygen diffusion via computer simulations. We use high-angle annular dark field (HAADF) imaging in a scanning transmission electron microscope (STEM) to image ion tracks, formed in Gd_2_Ti_2_O_7_ by irradiation with swift heavy 2.2 GeV Au ions, to investigate associated strain in the ring region. We use molecular dynamics (MD) simulations to model ion tracks to complement the experimental observations. We show that cation disorder leads to high oxygen diffusivity compared to ordered pyrochlore structure. In view of the many recent reports of enhancing oxygen diffusivity via tensile strain induced by heterointerfacing^15^,^16^, using density functional theory (DFT) calculations, we show that the high conducting defect-fluorite structure can be stabilized via tensile strain, and predict that oxygen diffusivity can be strain-enhanced by synthesizing pyrochlores as defect-fluorites via ion tracks or as thin-films. Thus this realization that control over the defect-fluorite structure can be possibly gained via tensile strain opens up new avenues for materials design for fast-ion conductor applications.

## Results

### Ion-track ring structure

**Figure 1:**
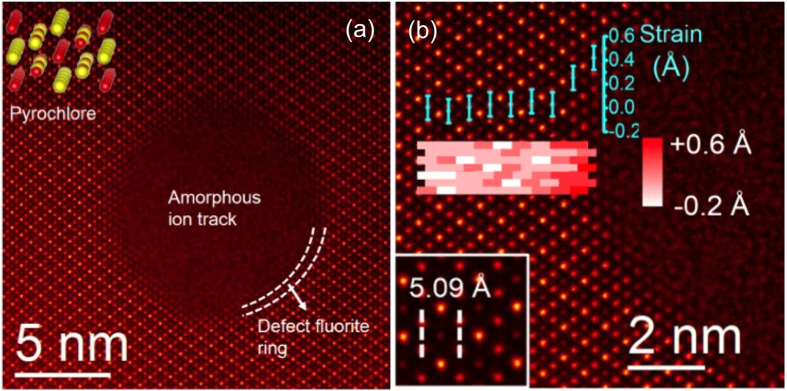
Ion track from STEM imaging. (**a**) A HAADF micrograph of an ion track in Gd_2_Ti_2_O_7_ created by 2.2 GeV Au ion showing section of defect-fluorite region (~1 nm thick) between pyrochlore matrix and amorphous region, indicated by the dotted lines. A pyrochlore atomic model with a [110] zone axis is shown to explain the atomic arrangement. Gd and Ti atoms are represented in yellow and red respectively. (**b**) Magnified image of the ion track. The bottom left inset shows the HAADF image of the bulk Gd_2_Ti_2_O_7_, oriented in [110] direction indicating the interatomic Gd-Ti distance. A strain map relative to the bulk value overlaid on the analyzed region is shown suggesting higher strain closer to the amorphous track/defect-fluorite region. Average strain in the atomic column emphasizes strain increase in the defect-fluorite region. Measurement error bar is ±0.1 Å.

A HAADF image of an ion track created by 2.2 GeV Au ion irradiation in a [110] projected Gd_2_Ti_2_O_7_ crystal is shown in Fig. 1a. The core of the track, ~8 nm in diameter, is amorphous in nature. The more interesting feature is observed at the transition between the pyrochlore (matrix) and the amorphous track/core, where a ~0.5 nm thick disordered crystalline transition region with defect-fluorite structure is seen surrounding the amorphous ion track. A section of the peripheral region is highlighted in Fig. 1a. Atomic model for pyrochlore (inset) along the [110] zone axis is shown in the inset to indicate the orientation of the crystal lattice in the HAADF image.

A detailed atomic structural analysis is presented in Fig. 1b, showing a magnified HAADF image. The inset in the left bottom corner shows the HAADF image of the bulk pyrochlore where the Gd-Ti interatomic distance along the [100] direction is 5.09 Å, which is half of the bulk lattice parameter of Gd_2_Ti_2_O_7_. A key observation from Fig. 1b is the presence of lattice expansion/tensile strain in the ring structure. The lattice expansion/strain shown in the figure is measured by comparing the atomic distances in the crystalline structure around the ion track with the atomic distances in the bulk Gd_2_Ti_2_O_7_ crystal. A strain map overlaying the corresponding atomic columns is shown as we progress from bulk towards the amorphous track. Based on the color bar, the strain in the bulk is almost zero, as expected. However, it gradually increases as we progress from bulk pyrochlore to the defect-fluorite ring structure. To further visualize this observation, a plot consisting of strain values obtained from the strain map averaged over parallel planes towards the ion track is also shown in Fig. 1b. This plot shows that, while the strain in the bulk pyrochlore (away from the ion track) is almost zero, it increases in the pyrochlore structure in the immediate vicinity of the ion track, and becomes significantly higher in the defect-fluorite region, illustrating the presence of tensile strain in the ring region compared to bulk pyrochlore.

**Figure 2:**
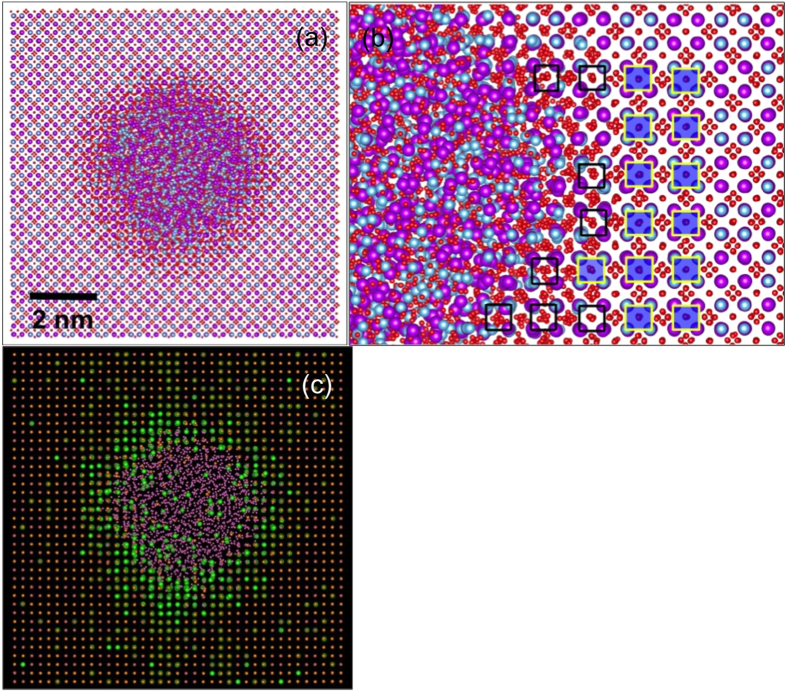
Ion track in MD simulation. (**a**) MD simulated ion track in Gd_2_Ti_2_O_7_ after 272 ps. (**b**) A close up snapshot of the ring region showing disordered defect-fluorite region highlighted via unfilled squares. The ordered region in the bulk is highlighted by filled squares. (**c**) Strain environment around the ion-track. The atoms under higher tensile strain are represented in bright green whereas those under minor or no strain are represented in orange. The atoms in amorphous region are represented in pink. The atoms in disordered, defect-fluorite ring region are under tensile strain.

Atomistic modeling of track formation using MD simulations reveals similar underlying features of ion tracks. Figure 2a shows an ion track obtained by introducing a thermal spike, described by a Gaussian energy-deposition profile from the electrons to the atomic structure that leads to local melting, quenching and restructuring over tens to hundreds of picoseconds. The center of the ion track is fully amorphous and, moving outward, is followed by a transition ring region and the surrounding pyrochlore bulk. To highlight the crystalline disordered ring region, a snapshot of the track is shown in Fig. 2b. The ordered pyrochlore bulk is highlighted by filled squares formed on the ordered *A* and *B* cation lattice. (Due to their ordering these squares cover either only *A* or only *B* cations.) As we approach towards the track, the cations begin to disorder. This cation disordering is highlighted by unfilled squares formed by black lines. Now, the squares do not contain only one type of cation; they rather consist of both, illustrating the disorder on the cation lattice. Thus, while the ring region maintains crystallinity, it loses its cation ordering. Similarly, the oxygen sublattice also loses its ordering and becomes fully disordered.

Figure 2c shows the strain environment in the simulated ion track represented by color-coded atoms. We calculated strain by measuring cation-cation interatomic distance with respect to that in bulk pyrochlore, similar to our experimental criteria above. While compensating for the atomic vibrations, those cation that have interatomic distance of up to 1.5% tensile strain are colored in orange. The cations that have more than 1.5% tensile strain are colored in bright green. The amorphized region is shown in pink. For clarity, oxygen atoms are not shown. We find that the atoms that have relatively smaller strain (i.e., <1.5%) primarily represent the bulk pyrochlore lattice away from the ion track. More interestingly, the atoms that are nearer to the track covering the ring region are under larger tensile strain. Some of these atoms have tensile strains as high as 6% in the ring region. Thus, in view of Fig. 2b,c, atomistic simulations, in good agreement with experimental observations, reveal that the atoms in the ring region are disordered and are under significant tensile strain.

Since the amorphous ion track and the surrounding disordered crystalline phase evolution occur by superheating to several thousand degree during initial melting followed by generation of shock wave and ultimately quenching of the track region over tens of picoseconds, one may intuitively expect compression in the disordered defect fluorite region due to possible volume expansion of the amorphous phase. However, the elements of this rather surprising result have been previously observed in ion-track structure investigations of CeO_2_. In the swift heavy ion radiation experiments on CeO_2_ by Takaki *et al.*^17^ and Yasuda *et al.*^18^, it was observed that large numbers of interstitials were outwardly emitted from the ion track towards the surrounding bulk during this process. These interstitials were identified as interstitial dislocation loops formed in the vicinity of the ion tracks. This depletion in the atomic density inside the ion track followed by quick quenching leading to shrinkage in the amorphous region could possibly explain the tensile strain observed by the atoms in the ring region.

### Pyrochlore to defect-fluorite phase transition

**Figure 3:**
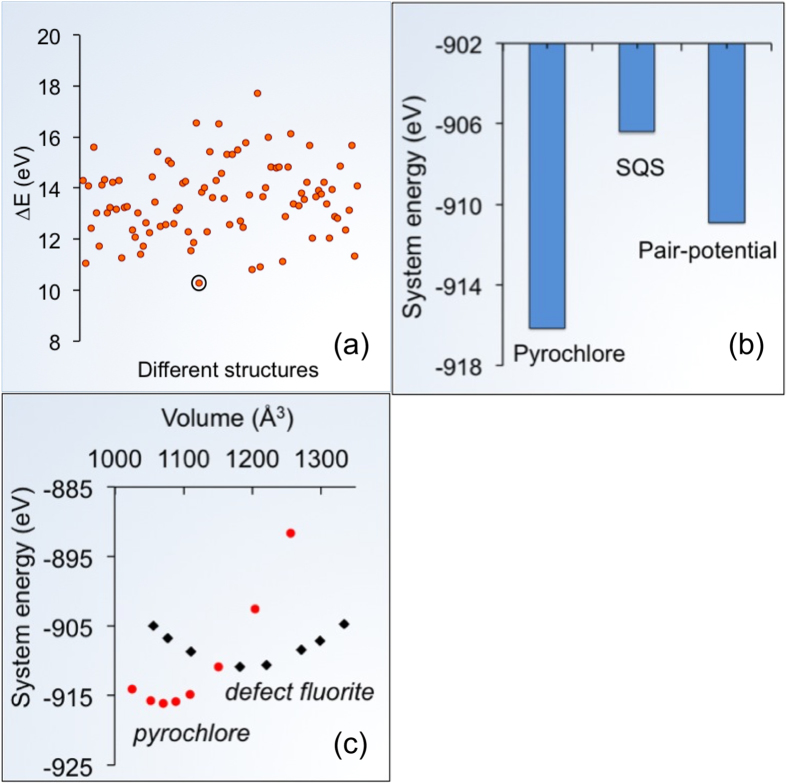
DFT defect-fluorite structure and relative energy with pyrochlore structure. (**a**) Relative energy of 100 different randomly generated defect-fluorite structures with respect to pyrochlore structure. (**b**) DFT computed system energies of pyrochlore, SQS and pair-potential-generated defect-fluorite structure. (**c**) Equation-of-state DFT energy comparison of the pyrochlore and defect-fluorite structures.

Now, we focus on the underlying mechanism of structural transition between pyrochlore and defect-fluorite. Using atomistic calculations, Rushton *et al.*^19^, had previously predicted that the transformation of pyrochlore to defect-fluorite structure could be possibly associated with lattice expansion. It is likely that the presence of the tensile strain captured in our HAADF images and MD simulations may have stabilized the defect-fluorite structure during epitaxial recrystallization that occurs during the track formation process. To elucidate the stability of this structural transition, we use DFT calculations in conjunction with static pair-potential simulations. In the pyrochlore structure (space group #227), *A* and *B* cations are found at *16c* and *16d* sites, respectively, and the oxygen sites are located at *48f* and *8a*, in addition to vacant *8b* sites. In contrast, the nature of cations on cation sites and the occupancy (occupied or not) of oxygen sites are not defined in the defect-fluorite structure, as the atoms are disordered. Therefore, we first predict the lowest energy defect-fluorite crystal structure to locate the atomic positions. Since there are infinite possible atomic arrangements, we use static pair-potential calculations that are computationally less expensive than DFT to predict the lowest energy structure, and then use this structure as an input for DFT calculations. Figure 3a shows the relative energy of 100 such disordered structures with respect to the pyrochlore structure of Gd_2_Ti_2_O_7_. These structures are created by randomly placing both *A* and *B* cations, and oxygen anions in the fluorite supercell lattice. We restrain our supercell size to 88-atom pyrochlore unit cell. The structures are relaxed using the LAMMPS code^20^. The lowest energy structure encircled in Fig. 3a is used as the defect-fluorite structure for further calculations. The atomic positions are given in the supplemental section.

Other techniques such as special quasirandom structure (SQS) can also be used to determine lowest energy structures. Jiang *et al*^21^ previously used this technique to predict an 88-atom SQS disordered structure for studying variety of defect-fluorite compositions, including Gd_2_Ti_2_O_7_. A comparison of the DFT calculated system energies of pyrochlore, SQS and pair-potential structures is given in Fig. 3b. As expected, the fully ordered pyrochlore has the lowest energy. The energy difference with respect to the pyrochlore structure for pair-potential and SQS structures are 0.65 and 1.21 eV/f.u, respectively. We find that our calculation of 1.21 eV/f.u. for the SQS structure agrees fairly with ~1.4 eV/f.u. given by Jiang *et al.*^21^. Since the pair-potential structure is lower in energy than the SQS structure, we perform the rest of the calculations using the pair-potential structure.

Now we return to the stability of the pyrochlore to defect-fluorite structure transition. The larger interatomic distances from the HAADF image suggest that the defect-fluorite structure has a larger volume than the pyrochlore structure. We use DFT calculations to elucidate the stability of this structure transition as a function of volume. Figure 3c shows the equation-of-state analysis for the stability of the two structures. The data points are obtained by calculating the system energy, while hydrostatically straining the structures. The plot shows that while defect-fluorite is unstable with respect to pyrochlore for the volume representative of the equilibrium pyrochlore, the stability trend gradually changes and pyrochlore becomes unstable at larger volumes compared to defect-fluorite. Thus, these calculations show that volume increase/tensile strain could be the reason for the stability of the defect-fluorite structure in the ring region around the ion track.

### Strain-engineered oxygen diffusion

**Figure 4:**
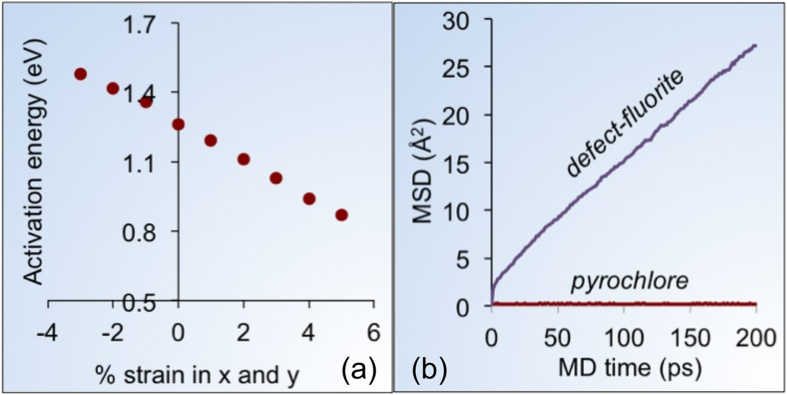
Tensile strain affected oxygen migration barriers. (**a**) *48f-48f* oxygen activation energy (eV) in pyrochlore structure as a function of planar strain applied in *x* and *y* directions. (**b**) Mean square displacement (MSD) of oxygen in defect-fluorite and pyrochlore structure. Higher MSD slope in defect-fluorite than pyrochlore structure reveals higher oxygen diffusivity.

In addition to tensile strain contributing to the structural stability of the transition, other elements of the structure can also be activated by strain, such that pyrochlore materials could be desirably ‘functionalized’ for technological applications. Recent research has shown that oxygen diffusivity increases significantly when a material is tensile strained by heterointerfacing^15^,^16^,^22^. This phenomenon has been shown in multilayer and thin-film materials, triggered by an experimental work on YSZ | SrTiO_3_ interface^15^. Simulation studies have also predicted lower oxygen migration barriers under tensile strain. Here, using atomistic simulations, we strain the pyrochlore structure to understand the oxygen vacancy activation behavior. In these simulations, we applied planar strain and calculated the activation energy of the *48f* -*48f* oxygen diffusion^6^. Using this diffusion path, our calculated activation energy at zero strain is 1.26 eV, in agreement with a previous study^6^. The effect of strain on the activation energy is shown in Fig. 4a, which reveals a consistent decrease in activation energy with increase in tensile strain, thus emphasizing that strain could be used to tailor oxygen conductivity in these materials.

Most of the interface-induced, strain-dependent oxygen diffusivity work has largely remained focused on fluorite and perovskite materials, whereas pyrochlore materials have remained elusive to such scrutiny. Breaking the vacancy ordering in the defect-fluorite structures via tensile strain opens a pathway for easier oxygen diffusion. This is evident in our MD simulations (see Fig. 4b) performed at 2000 K for 200 ps on pyrochlore and defect-fluorite structures. Both these simulations are performed using 4 × 4 × 4 supercell containing 5,632 atoms. Oxygen diffusion is captured via mean square displacement (MSD) which is related to diffusivity (D) with the relationship, MSD = 6Dt, where t is time. While the defect-fluorite structure shows high oxygen diffusivity evidenced by the slope of the MSD curve, the pyrochlore structure shows minimal oxygen diffusion. These contrasting results show significant difference in oxygen diffusion, and elucidate that breaking the atomic symmetry is the key. Similar MSD observations were made previously by Wilde and Catlow^23^ and by others^24^.

## Discussion

The above results show that tensile strain is a common denominator in structural transition and in lowering oxygen migration barriers. Since most of the A_2_B_2_O_7_ compositions that have low migration barriers do not exist as defect-fluorites but rather as ordered pyrochlores^6^,^8^, tensile strain could be used as a design parameter in stabilizing them as defect-fluorite structures. The applied strain would thus have a two-pronged effect in also reducing the oxygen migration barriers.

Ion-beam modification and implantation are proven methods in functionalizing semiconductors and complex oxides for device applications^25^,^26^. Here, irradiation-induced ion tracks, which can be created with MeV to GeV ions^25^,^26^ promise to open up a new avenue for structural and defect engineering. While there are other pyrochlore compositions such as Gd_2_Zr_2_O_7_ that show structure transition only from pyrochlore to defect-fluorite without the formation of the amorphous core^11^, posing a case for the use of Gd_2_Zr_2_O_7_ instead of Gd_2_Ti_2_O_7_, our calculations reveal that oxygen migration barriers are lower in Gd_2_Ti_2_O_7_ than Gd_2_Zr_2_O_7_, thus supporting the choice of Gd_2_Ti_2_O_7_ and its ring structure for ion-transport applications. An MSD comparison of the two defect-fluorite systems is shown in Figure S1 that shows higher diffusivity in Gd_2_Ti_2_O_7_ than Gd_2_Zr_2_O_7_. During ion-implantation, the diameter and length of the ring structure can also be varied by changing the energy of the impinging ions, and the composition of the target materials. These parameters thus provide even greater control over the structure of the ion tracks.

Besides ion implantation, heterointerfacing is another possible route in stabilizing the defect-fluorite structure. Some recent studies have gained traction in synthesizing pyrochlore thin films^27^, however, the oxygen conductivity measurements in these thin films have not yet been explored. Therefore, there remain opportunities to gain significant ground in achieving high oxygen conductivities via heterointerfacing. In addition, trivalent dopants added to fluorite materials, which often segregate to interfaces and reduce oxygen conductivity by forming complex-defect associates^22^, are absent in these materials. Thus, the free availability of oxygen vacancies in defect-fluorite structure could be an additional factor in advancing the strain-engineered pyrochlore materials.

In conclusion, we have used DFT calculations, MD simulations, ion-radiation experiments and HAADF imaging to elucidate the stability of the defect-fluorite ring structure around the amorphous core of the ion track. Our DFT calculations show that under track formation the ordered pyrochlore can undergo structural transition to disordered defect-fluorite, which is stabilized by tensile strain. The presence of tensile strain is observed in HAADF images and in our MD simulations. The results suggest that this strain occurs during the complex track formation process from a swift-heavy ion-induced thermal spike, which leads to melting, ejection of atoms from the core and rapid quench cooling. Using MD simulations, we also show that the defect-fluorite structure has very high oxygen diffusivity due to breaking of the atomic ordering. In addition, we show that tensile strain lowers oxygen migration barriers. Thus in view of materials design, we suggest that tensile strain introduced either by ion-irradiation or by heterointerfacing could be practically used to stabilize the high conducting defect-fluorite structure for fast-ion conductor applications.

## Methodology

### Ion irradiation

The polycrystalline samples were prepared by the sol-gel method, followed by a sintering process at 1875 K for 50 hrs in air. The samples were polished down to ~50 μm thickness to ensure that irradiated ions will pass through the material. The samples were further irradiated by swift heavy 2.2 GeV Au ions using X0 beam line of UNILAC linear accelerator in the GSI Helmoltz Center for Heavy Ion Research in Darmstadt, Germany. For creating isolated ion tracks, the fluence was kept at 5 × 10^10^−1 × 10^11^ ions/cm^2^.

### STEM Imaging

HAADF imaging was performed on various samples in a 5^rd^ order aberration corrected scanning transmission electron microscope (Nion UltraSTEM200) operating at 200 KV. Detector with an inner angle of 65 mrad was used to collect electrons for HAADF imaging. The electron probe current used in the experiment was 28 pA. The plan-view samples for STEM analysis were prepared by conventional mechanical thinning, precision polishing and ion-milling in liquid N_2_ environment.

### Computational

DFT calculations are performed using the Vienna *Ab Initio* Simulation Package^28^ (VASP). In particular, the projector-augmented wave (PAW) method with plane waves up to the energy cutoff of 500 eV is used, and the exchange-correlation is evaluated by the generalized-gradient approximation (GGA) using the Perdew-Burke-Ernzerhof (PBE) function^29^. All computations are based on 88 atoms. Integrations over the Brillouin zone were carried out using the Monkhorst-Pack scheme with a *k-point* sampling of 2 × 2 × 2. All relaxations are done until the forces were smaller than 0.01 eV/Å. After the tests for plane-wave energy cutoff and *k-point* sampling, the numerical error is less than 1 meV/atom. The Gd *4f* electrons are described by the Hubbard*-U* correction with spin-orbital coupling effects considered in which *U*_*eff*_* *= 6.9 eV is used based on the previous similar work^30^. The calculated lattice parameter for bulk Gd_2_Ti_2_O_7_ is 10.23 Å in typical DFT agreement with the experimental value of 10.18 Å.

The ion-track MD simulations are performed using the DL-POLY code^31^. A system size of 35 × 35 × 15 unit cells containing a total of 1,617,000 atoms is used. The Buckingham type interatomic potential from Pirzada *et al.*^6^ is used. Before introducing the thermal spike, the system is first annealed at 300 K for 20 ps. The thermal spike is introduced by giving kinetic energy to atoms in random directions to mimic the radial energy deposition profile transferred from the electrons to the atoms due a 2.2 GeV Au ion (27 keV/nm energy loss) passing along the z-direction, i.e., into the plane of the page. The thermal spike is described by a Gaussian profile with a 2 nm standard deviation, in accordance to similar calculations 12 keV/nm energy loss irradiation of Gd_2_Ti_2_O_7_^10^. The simulation is done at constant volume and run for 272 ps, and the outermost 0.5 nm thick layer of the simulation box is coupled to a 300 K heat bath in x and y boundaries.

## Additional Information

**How to cite this article**: Aidhy, D. S. *et al.* Fast ion conductivity in strained defect-fluorite structure created by ion tracks in Gd_2_Ti_2_O_7_. *Sci. Rep.* **5**, 16297; doi: 10.1038/srep16297 (2015).

## Supplementary Material

Supplementary Information
