# Supplementary material for: Fast ion conductivity in strained defect-fluorite structure created by ion tracks in Gd2Ti2O7
Source: Sci Rep. 2015 Nov 10;5:16297. doi: 10.1038/srep16297 (PMC4639808; doi:10.1038/srep16297)
Supplement: Supplementary Information [file srep16297-s1.pdf]

Fast ion conductivity in strained defect-fluorite structure created by ion tracks in $Gd_2Ti_2O_7$

Dilpuneet S. Aidhy,[1*] Ritesh Sachan,[1] Eva Zarkadoula,[1] Olli Pakarinen,[1] Matthew F. Chisholm,[1] Yanwen Zhang[1,2] and William J. Weber[2,1]

[1]Materials Science and Technology Division, Oak Ridge National Laboratory, Oak Ridge, TN 37831

[2]Materials Science and Engineering, University of Tennessee, Knoxville, TN 37996

Supplemental section.

Pair-potential atomic structure for DFT calculations based on VASP POSCAR format

```
A2B2O7-pair potential structure
  1.00000000000000
   10.7057263856707170    0.1694045628923825   -0.3757171814431654
   -0.2683304549073293   10.5063972968573083   -0.3626698640182821
   -0.4093416580921933   -1.0612120147165280   10.5563859560874231
  O   Ti   Gd
   56   16   16
Direct
  0.1254065444880517  0.1517767188827154  0.1252192059662782
  0.3895413246683580  0.1375839316553509  0.1241922411489867
  0.1442640785082031  0.3933558337708462  0.1756386946361855
  0.1463379939232248  0.2261540550597347  0.3656778825069111
  0.3305734744185114  0.0881020227438570  0.4500628750382263
  0.3829082362621640  0.4118413672760788  0.3646392680812318
  0.1125044650886994  0.0929433332632502  0.5846370261597434
  0.3771358840558462  0.0947590388224464  0.7105959459397894
  0.1192097850011082  0.3480181774414141  0.6222988102466390
  0.3631540273501880  0.3216740071992447  0.6241138905889864
  0.1319138230990792  0.1193139381016266  0.8644630835123669
  0.5982352323580910  0.1366079647928165  0.8639183931695135
  0.1356538915269754  0.3767634137182067  0.8914241611352099
  0.4227214547765298  0.3414744429345601  0.8871183851970144
  0.1395157512234323  0.6494129159197849  0.1462881995604620
  0.3802202997074699  0.4318955368463745  0.1269557940893455
  0.1263687584979914  0.8959821740563112  0.1323805836565749
  0.4072956370558958  0.8666338269513879  0.1389750863161240
```

0.1645295752398056	0.5634450471979678	0.4082704566469925
0.3640203469055149	0.6648120054656732	0.2920601706632914
0.0927805737672319	0.8094397078908936	0.3769423847716363
0.1454859514976555	0.6225437186363710	0.6463413887847469
0.4202100449144527	0.6101798699747535	0.6825672128378203
0.0575335086115062	0.8323955765657323	0.6192310977973734
0.3054535629671384	0.8411902956594309	0.5611722129078637
0.1052323615228856	0.6208765905328361	0.8804254782246231
0.3751482040941495	0.5956106703377879	0.9366974832928270
0.1264771679520821	0.8598295210867118	0.8680704932708592
0.3785301864819229	0.8655225087915235	0.8531425463653707
0.6502624973923518	0.1346016529973080	0.1369287208630126
0.8944541032966578	0.1332113859559256	0.1362060400022616
0.6291552683773872	0.3991006122983729	0.0984476389616219
0.9017372691554816	0.3858362359791447	0.1467364459287188
0.7585508819179023	0.0728134725429928	0.3748237488505166
0.6415046769787005	0.3222423184181678	0.4165218540406134
0.9134495198547201	0.2942760398483761	0.3782130319195290
0.5752571376461448	0.1170183797459896	0.5968262600111152
0.8500389719989321	0.1212331895616016	0.6003117124491401
0.8468859784885759	0.3673684008616583	0.6119655354713228
0.8649932204853327	0.1078525858417976	0.8627593782071492
0.6182837269659237	0.3707796831604442	0.7241606858471031
0.8626065837688416	0.3852603183340985	0.8675361675353377
0.5674630573105770	0.6436673696646993	0.1232209609149823
0.7007289812961953	0.8859637340175337	0.1037379444003696
0.8843769921306027	0.6795173166813707	0.1144772622609983
0.6316474891965361	0.6833789838984626	0.3654894236914742
0.8574301053248801	0.5982627605687852	0.4116321953985467
0.4998874357213457	0.9089776204820804	0.3816325198099100
0.9145464106393165	0.9178738022519830	0.2590369961904308
0.6691597089375318	0.5856168926615983	0.6149080349129813
0.9017068239328675	0.6037731632616006	0.6960358921422525
0.5429561528500264	0.8713850112233438	0.6371173911676079
0.7984276732312425	0.8497381813800319	0.5669325540541533
0.6240062206353756	0.8251997353815620	0.8666198032713024
0.7233779023040074	0.5923762634474065	0.8960774567127537
0.8878748673982445	0.8172538387159145	0.8910128171447169
0.0199663879861653	0.2687877958838662	0.2257331768740535
0.2150797862259241	0.2153519294369156	0.5288808563822907
0.0324416798912062	0.7815652282844269	0.2077381728948395
0.2692891883330440	0.5071323847464837	0.2820609586984903
0.0309869354833665	0.7163398576052945	0.7631820458767898
0.1738305279120676	0.7349432868574916	0.5142044060754083
0.7826270888781480	0.0272801389628086	0.2089539425048707
0.4551537628657292	-0.0098303784756803	0.5333704017052163

0.4925446070203015	0.2248597996782799	0.7530085268223001
0.7865655955544998	0.2416807157544149	0.4729636794135283
0.4813962161243637	0.4941233881896401	0.0199446687783331
0.4923345374754999	0.7593810007395161	0.2589348287115303
0.7611135125000175	0.7567622305669619	0.9672071697381618
0.4845535247889562	0.7702555738021863	0.7487032697505744
0.7629085706241281	0.4751882406089312	0.7502816622670588
0.7409752291211665	0.6857407435559089	0.5090362174160787
0.0100649651655112	0.0088583952724120	0.9767206084422272
0.2668267341268351	0.0428689760561800	0.2441838266267959
0.2501922480557114	0.2621160092251627	0.0089215405856349
0.9433522556431569	0.9588269289700098	0.4784677534515148
0.9919931648729057	0.2384984390094958	0.7483503088775945
0.2168122027798533	0.9695672631806320	0.7314951568114837
0.0175575818956391	0.5002515898056705	0.0199352765959791
0.2505967659656666	0.7572555772512425	0.0035286701354328
0.9936434126596739	0.4805785600196401	0.5236374363584657
0.2616287134288419	0.4782874556508819	0.7631454396705352
0.5257381992512560	0.9798191355807644	0.0143468902683595
0.5237193342964154	0.2900808120391574	0.2285713759844611
0.7635770328082599	0.2484613009334790	0.0014296163708994
0.7195795024816387	0.0084143422806450	0.7111913963883361
0.7511465258786204	0.5643486643163498	0.2127960212098969
0.5065580714398369	0.4478967530705649	0.5523414077039254

Figure S1

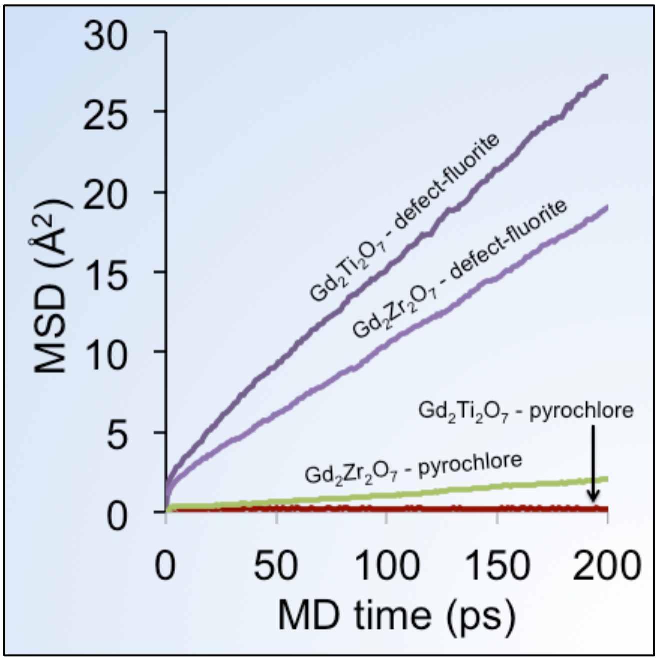

Figure S1. Mean square displacement (MSD) of $Gd_2Ti_2O_7$ and $Gd_2Zr_2O_7$ for their corresponding defect-fluorite and pyrochlore structures obtained from MD simulations. The simulations show that oxygen diffusivity in the defect-fluorite structure of $Gd_2Ti_2O_7$ is greater than that of $Gd_2Zr_2O_7$.
